# Interventions for the management of Pain and Sedation in Newborns undergoing Therapeutic hypothermia for hypoxic-ischemic encephalopathy (IPSNUT): protocol of a systematic review

**DOI:** 10.1186/s13643-022-01982-9

**Published:** 2022-05-23

**Authors:** Pyrola Bäcke, Matteo Bruschettini, Ylva Thernström Blomqvist, Emma Olsson

**Affiliations:** 1 University Hospital, Neonatal Intensive Care Unit, Uppsala, Sweden; 2 Department of Women’s and Children’s Health, Uppsala University, Uppsala, Sweden; 3 Department of Pediatrics, Lund University, Lund, Sweden; 4 Cochrane Sweden, Research and Development, Skåne University Hospital, Lund, Sweden; 5 Department of Pediatrics, Faculty of Medicine and Health, Örebro University, Örebro, Sweden; 6 Faculty of Medicine and Health, School of Health Sciences, Örebro University, 701 82 Örebro, Sweden

**Keywords:** Therapeutic hypothermia, Neonatal, Asphyxia, Hypoxic-ischemic encephalopathy, Cooling, Pain scale

## Abstract

**Background:**

Clinical research has shown that therapeutic hypothermia after neonatal hypoxic-ischemic injury improves survival without disability. There is no consensus regarding pain relief or sedation during therapeutic hypothermia in newborns; however, therapeutic hypothermia seems to be associated with pain and stress, and adequate analgesia and sedation are central to maximize the effect of therapeutic hypothermia. Pain needs to be adequately managed in all patients, especially the newborn infant due to the potential short- and long-term negative effects of inadequately treated pain in this population.

**Methods:**

We will perform a systematic review of pharmacological and non-pharmacological interventions for the management of pain and sedation in newborn infants undergoing therapeutic hypothermia for hypoxic-ischemic encephalopathy. We will include randomized, quasi-randomized controlled trials and observational studies. The use of pharmacological or non-pharmacological interventions will be compared to other pharmacological and or non-pharmacological interventions or no intervention/placebo. The primary outcomes for this review will be analgesia and sedation assessed with validated pain scales, circulatory instability, mortality to discharge, and moderate-to-severe neurodevelopmental disability. We will search the following databases: CINAHL, ClinicalTrials.gov, Cochrane Library, Embase, PubMed, Scopus, and Web of Science. Two independent researchers will screen the records for inclusion, extract data using a data extraction form, and assess the risk of bias in the included trials.

**Discussion:**

The result of this review will summarize the knowledge regarding the management of pain and sedation in infants treated with therapeutic hypothermia and potentially provide clinicians with guidance on the effective and safe methods.

**Systematic review registration:**

PROSPERO CRD42020205755

**Supplementary Information:**

The online version contains supplementary material available at 10.1186/s13643-022-01982-9.

## Background

### Description of the condition

Peripartum asphyxia affects three to five newborns per 1000 births, with subsequent moderate to severe hypoxic-ischemic encephalopathy (HIE) in approximately two to three per 1000 live births [1, 2]. In order to classify HIE, the Sarnat Grading System is most commonly used with three stages ranging from mild and moderate to severe. The grading is based on the clinical evaluations of the level of consciousness, neuromuscular control, reflexes, autonomic function, and seizures [3]. Clinical research has shown that therapeutic hypothermia (TH) improves survival without disability for infants with moderate to severe HIE [4, 5]. Therapeutic hypothermia should be initiated as soon as possible within the first 6 h after birth and maintained for 72 h [2]. The brain can be cooled with whole-body or selective head cooling; both methods seem to be equally effective but whole-body cooling is the most commonly used [2]. The infant is then placed on a servo-controlled cooling mattress that maintains the infants’ temperature at 33.5 °C. Infants with HIE undergoing TH need adequate management for pain and sedation because of their clinical condition and the need for intensive care, such as cooling and respiratory support.

The TH seems to be associated with pain and stress [6], and adequate analgesia and sedation are central to maximize the effect of TH [7]. Infants receiving TH are also exposed to stress and frequent painful procedures [8]. Additionally, newborn infants with a high risk of neurological impairment are subjected to a greater number of painful procedures during their first days of life than newborn infants with lower risk, and despite this, the high-risk infants usually receive less analgesics such as opioids [9]. Painful experiences in newborns can result in altered pain responses later in life [10] and can also impair the infants’ brain development [11]. Assessing pain in infants that are not only post-asphyxiated but also treated with TH is a difficult task due to their neurological deficiencies. Although there are no pain scales validated for pain assessment during TH, pain scales validated for neonates are often used in the clinical setting for infants during TH [7].

Therapeutic hypothermia causes a redistribution of regional blood flow, which may impact both drug distribution and clearance. It has also been associated with decreased glomerular filtration rate in animal studies and may therefore decrease renal excretion of drugs in humans [12]. Since the effects of sedative and analgesic treatment during TH, on both short- and long-term outcomes, are unclear [13] and the knowledge that TH can affect pharmacokinetics [14], caution is often recommended. Therapeutic hypothermia leads to longer serum clearance of morphine, fentanyl, and midazolam [15], and morphine infusions higher than 10 mcg/kg/h might be associated with toxic serum levels in some infants [16]. In addition to the abovementioned difficulties, the infants undergoing TH will in many cases have suffered hepatic and renal injuries due to asphyxia impacting how the infant will handle drugs that are given [12]. In addition, opioids might decrease gastrointestinal motility and thus increase the risk for perforation [17].

### Description of the intervention

Morphine is an analgesic and sedating drug frequently used in infants undergoing TH. During TH, morphine’s affinity for the μ opioid receptor is reduced, rendering it less effective; however, since the clearance of morphine is lower in infants, accumulation can occur if higher doses are used [18]. Adverse effects of morphine include miosis, priuritus, respiratory depression, constipation, urinary retention, and hypotension [19]. Fentanyl is used in neonatal care for severe procedural pain and prolonged pain [20] and during mechanical ventilation [21]. In adults, fentanyl is mainly cleared by hepatic biotransformation via cytochrome P450 (CYP) 3A4. In neonates, this enzyme has much lower activity during the first weeks of life, before increasing to values equal to the adult population [20]. The half-life of fentanyl shows an inter-individual variation in the neonate, and TH can lead to a 25% increase in plasma concentrations [12].

Midazolam causes sedation, amnesia, and muscle relaxation but not analgesia. Midazolam undergoes hydroxylation by CYP-450 in the liver and is eliminated by the kidneys. The elimination of midazolam is reduced in newborn infants [19], and the use of midazolam in adults during TH resulted in a fivefold increase in the serum levels [12]. Adverse effects of midazolam include hypotension and neurological abnormalities [22].

In contrast with opioids, alpha-2 agonists such as clonidine do not cause respiratory depression. Clonidine can also reduce opiate and benzodiazepine needs and also has analgesic properties. Clonidine undergoes CYP2D6-mediated hydroxylation and is eliminated by the kidneys. Clearance in infants is reduced because of pathway immaturity or renal disease. The adverse effects of clonidine include hypotension, rebound hypertension, atrioventricular block, and bradycardia [19]. Dexmedetomidine is a selective alpha-2 adrenergic agonist that also provides analgesia and sedation. Similar to clonidine, it has little impact on the respiratory drive and has been tested during TH [23].

Paracetamol is a non-opioid, central-acting analgesic used in the treatment of mild to moderate pain in neonatal care [19]. It is widely used in infants and children to treat fever and/or pain whereas in very preterm infants, it can also be administered for the management of patent ductus arteriosus [24]. Paracetamol is primarily metabolized in the liver, and metabolites are mainly cleared renally. Paracetamol also has opioid-sparing properties. The main adverse effect is hepatotoxicity caused by the metabolite *N*-acetyl-p-benzoquinone imine (NAPQI). NAPQI drains the liver of glutathione which acts as an antioxidant and directly damages the cells in the liver at the mitochondrial level [19].

Non-pharmacological interventions for the treatment of pain are commonly used in neonatal care partly because of their safety and their efficacy for mild pain. Non-pharmacological interventions also provide the infant’s parents with the possibility of being included in the infant’s pain care, thus facilitating parental bonding. Examples of these interventions are swaddling, sweet solutions, facilitated tucking, and non-nutritive sucking [25].

### How the intervention might work

Morphine is the opioid most commonly used in neonatal care and is an agonist of the μ and k receptors [18]. It acts by binding to opiate receptors in the central and peripheral nervous systems exerting its analgesic effect by stimulating descending inhibitory pathways. Morphine is primarily metabolized in the liver, and the pharmacokinetics vary significantly in neonates [19]. Fentanyl and its derivatives interact with the μ opioid receptor [26]. Midazolam is a short-acting benzodiazepine exerting its sedative effect by binding to GABA receptors [19].

With the use of alpha-2 agonists such as clonidine, alpha-2 receptors in the central nervous system are activated resulting in a decrease in sympathetic activity [19].

Paracetamol’s analgesic effect is mediated by activation of descending serotonergic pathways, and there is also inhibition of prostaglandin synthesis (cyclo-oxygenase) and formation of an active metabolite that influences cannabinoid receptors [27].

Non-pharmacological interventions employ environmental and behavioral approaches by activating a “gate control mechanism” that prevents pain sensation from being carried to the central nervous system [25].

### Why is it important to do this review

Pain needs to be adequately managed in all patients, including the newborn infant. Painful procedures and inadequate pain management lead to both short- and long-term negative effects [10]. Despite the pain and stress associated with TH, pain management and sedation of the infant during TH have not been systematically assessed for what is the best practice and clinical practices vary widely [7]. Stress and unrelieved pain can impact the TH negatively, and a comprehensive synthesis is therefore needed to determine the best available evidence on pain relief and sedation in infants treated with TH. Of note, no systematic reviews have been conducted on this topic.

## Objectives

The aim of the study is to highlight the current state of evidence regarding pharmacological and non-pharmacological interventions for the management of pain and sedation in newborn infants undergoing TH for hypoxic-ischemic encephalopathy.

## Methods

We will conduct a systematic review using the standard methods of the Cochrane Neonatal Review Group. We will search for all randomized and quasi-randomized controlled trials. We will also include observational studies, as we expect to identify few randomized trials and to better explore potential harms. The methodology will follow the criteria and standard methods of the Cochrane Handbook [28] and the reporting guidelines by the Preferred Reporting Items for Systematic Review and Meta-Analyses for Protocols (PRISMA-P) (Additional file 1). Ethical approval is not needed for a systematic review. The review is registered in PROSPERO (registration number: CRD42020205755).

### Types of participants

Late preterm (i.e., 34–36 weeks’ gestational age) and full-term (i.e., more than 36 weeks’ gestational age) newborn infants undergoing any type of TH for hypoxic-ischemic encephalopathy.

### Types of interventions

We will include studies using any type of drugs or any type of non-pharmacological intervention used for the management of pain and/or sedation during TH. We will include any dose, duration, and route of administration. Pharmacological interventions include any opioids, e.g., morphine; alpha-2 agonists, e.g., clonidine; and benzodiazepines, e.g., midazolam. Non-pharmacological interventions include non-nutritive sucking, sweet solutions (oral glucose or sucrose), swaddling, musical therapy, therapeutic touch/massage, sensorial saturation, or acupuncture.

### Types of outcome measures

Primary outcomes are analgesia and sedation assessed with validated pain scales in the neonatal population (the Echelle Douleur Inconfort Nouveau-ne (EDIN) Scale, the COMFORTneo, Faces Pain Scale-Revised, the Neonatal Pain, Agitation and Sedation Scale (N-PASS), Pain Assessment Tool, the Astrid Lindgren and Lund Children’s Hospital’s Pain and Stress Assessment scale for Preterm and Sick Newborn Infants (ALPS-neo), the Neonatal Facial Coding System (NFCS), the CRIES (acronym for Crying, Requires oxygen, Increased vital signs, Expression, Sleepless) Scale [29, 30], circulatory instability requiring medical therapy (inotropes, vasopressors, and/or fluid boluses), mortality to discharge and neurodevelopmental disability, defined as cerebral palsy, developmental delay (Bayley Scales of Infant Development - Mental Development Index Edition II (BSID-MDI-II), Bayley Scales of Infant and Toddler Development - Edition III Cognitive Scale (BSITD-III), Griffiths Mental Development Scale - General Cognitive Index (GCI) assessment greater than two standard deviations (SDs) below the mean), intellectual impairment (intelligence quotient (IQ) greater than two SDs below the mean), blindness (vision less than 6/60 in both eyes), or sensorineural deafness requiring amplification.

The secondary outcomes are neonatal mortality; duration of hospital stay; days to reach full enteral feeding; analgesia assessed with neurophysiological measures such as NIRS (near-infrared spectroscopy) or GSR (galvanic skin response); focal gastrointestinal perforation; episodes of bradycardia (heart rate < 80 beats/min); signs of distress, e.g., heart rate > 100 beats/min; and neurodevelopmental disability.

### Search methods

We will search for studies in the following databases: CINAHL, ClinicalTrials.gov, Cochrane Library, Embase, PubMed, Scopus, Web of Science, and conference proceedings with no restrictions for time and language (search strategy is shown in Additional file 2). We will also perform a manual search through references in the found articles.

### Selection of studies

Two independent researchers will independently screen the titles and abstracts followed by a full-text screening using an online tool for the preparation of systematic reviews [31]. Disagreements will be solved by a third researcher or in discussion with the group, as proposed by the Cochrane Handbook [28].

### Data extraction and management

Data will be extracted using a data extraction form by two authors independently. Disagreements will be solved by a third researcher or in discussion with the group. The following data will be extracted:

- Administrative details (author(s), year of publication)
- Details of the study: design, type, duration, country and location of study, funding, informed consent, and ethical approval
- Details of participants: birth weight, gestational age, number of participants, and HIE severity
- Details of intervention and comparator: type of drug/non-pharmacological intervention and dosages
- Details of outcomes, as listed in types of outcome measures

Should any queries arise or in cases where additional data are required, we will contact the study investigators/authors for clarification.

### Assessment of risk of bias in the included studies

Two researchers will independently assess the risk of bias (low, high, or unclear) of all included studies using the Cochrane “Risk of Bias” tool. Any disagreements will be resolved by discussion in the group. The two researchers will independently assess the risk of bias (low, high, or unclear) of all included trials using the Cochrane “Risk of Bias” tool for the following domains:

- Sequence generation (selection bias)
- Allocation concealment (selection bias)
- Blinding of participants and personnel (performance bias)
- Blinding of outcome assessment (detection bias)
- Incomplete outcome data (attrition bias)
- Selective reporting (reporting bias)
- Any other bias

For the included observational studies, we will use the “Risk of Bias in non-randomized Studies – of Interventions (ROBINS-I) tool to assess the risk of bias [32]. Two researchers will independently assess the risk of bias (low, moderate, serious, critical, no information) of all included observational studies using the ROBINS-I tool for the following domains:

- Bias due to confounding
- Bias in the selection of participants for the study
- Bias in the classification of interventions
- Bias due to deviations from intended interventions
- Bias due to missing data
- Bias in the measurement of outcomes
- Bias in the selection of the reported result

In the confounding domain, we will take into account the following confounding factors: sex, Apgar score, severity of HIE, mode of delivery, and gestational age.

### Strategy for data synthesis

We will summarize all eligible studies in Review Manager 5.4. We will utilize the standard methodologies for meta-analysis as described in the Cochrane Handbook for Systematic Reviews of Interventions [28]. We will use the random-effect model and present all our results with 95% CI. We will calculate the RR, RD, and NNTB or NNTH if RD is significant, each with 95% CI, for categorical outcomes, and MD with 95% CI for continuous outcomes. For any outcomes where the included studies are not sufficiently homogeneous, or where insufficient data are available for meta-analysis, we will present a narrative synthesis, following the Synthesis without meta-analysis (SWiM) Guidelines [33]. We will not pool randomized studies and observational studies in the same analyses.

We plan to assess clinical heterogeneity by comparing the distribution of important participant factors between trials and trial factors (randomization concealment, blinding of outcome assessment, loss to follow-up, treatment type, co-interventions). We will assess statistical heterogeneity by examining the *I*^2^ statistic [28], a quantity that describes the proportion of variation in point estimates that is due to variability across studies rather than sampling error.

We will interpret the *I*^2^ statistic as described by Higgins 2019:

- < 25%: no heterogeneity
- 25 to 49%: low heterogeneity
- 50 to 74%: moderate heterogeneity
- ≥ 75%: high heterogeneity

We will consider statistical heterogeneity to be substantial when *I*^2^ is greater than 50%. In addition, we will employ the *χ*^2^ test of homogeneity to determine the strength of evidence that heterogeneity is genuine. We will explore clinical the variation across studies by comparing the distribution of important participant factors among trials and trial factors (randomization concealment, blinding of outcome assessment, loss to follow-up, treatment type, and co-interventions). We will consider a threshold *P* value of less than 0.1 as an indicator of whether heterogeneity (genuine variation in effect sizes) is present.

### Assessment of reporting biases

We will investigate publication by using funnel plots if at least 10 clinical trials are included in the systematic review [28, 34].

### Certainty of evidence

We will use the Grading of Recommendations Assessment, Development, and Evaluation (GRADE) approach, as outlined in the GRADE Handbook, to assess the certainty of evidence for the primary outcomes of this review (see above).

Two authors will independently assess the certainty of the evidence for each of the primary outcomes specified above, in the section types of outcomes. We will consider evidence from randomized controlled trials as high but downgrade the evidence by one level for serious (or two levels for very serious) limitations based upon the following: design (risk of bias), consistency across studies, directness of the evidence, precision of estimates, and presence of publication bias. We will consider evidence from observational studies as low but upgrade the evidence (one or two levels) based upon the following: large magnitude of an effect, dose-response gradient, and effect of plausible residual confounding. We will use the GRADEpro Guideline Development Tool to create a “summary of findings” table to report the certainty of the evidence for randomized studies and observational studies, respectively.

### Sensitivity analysis

We will conduct sensitivity analyses to explore the effect of the methodological quality of the trials, checking to ascertain if studies with a high risk of bias overestimate the effect of treatment.

Analysis of subgroups or subsets:

- Severity of HIE, based on the Sarnat staging
- High versus low dose of the intervention (thresholds will be set post hoc)
- By route of administration, e.g., enteral or intravenous

## Discussion

Two to three infants per 1000 live births are affected with moderate to severe hypoxic-ischemic encephalopathy (HIE) and could subsequently be treated with TH. Even though this treatment has been available since 2006 [35], there are still no guidelines for appropriate analgesia and/or sedation during TH. The negative effects of painful procedures during the neonatal period are known through extensive research, and it would seem that the already injured asphyxiated brain would benefit from a balanced approach between the pain and stress of being cooled and the potential negative effects of pharmacological treatments.

A brief literature search indicated that the number of current articles might be not too many. Therefore, we will include all pharmacological and non-pharmacological interventions that could affect pain or sedation in the infant. The result of this review might lead to a better understanding concerning managing pain and sedation during TH, which can result in new recommendations towards effective and safe pain and sedation for the infant during the treatment period. We also believe that the result might identify unexplored areas in this field where more research is necessary to develop strategies that will be able to provide clinicians with guidance.

## Supplementary Information

**Additional file 1.** PRISMA-P 2015 Checklist.

**Additional file 2.** Search Report.

## Data Availability

Not applicable

## Abbreviations

ALPS-neo: Astrid Lindgren and Lund Children’s Hospital’s Pain and Stress Assessment scale for Preterm and Sick Newborn Infants
CRIES: Crying, Requires oxygen, Increased vital signs, Expression, Sleepless
EDIN: Echelle Douleur Inconfort Nouveau-ne Scale
GRADE: Grading of Recommendations Assessment, Development, and Evaluation
GSR: Galvanic skin response
HIE: Hypoxic-ischemic encephalopathy
NAPQI: *N*-acetyl-p-benzoquinone imine
NFCS: Neonatal Facial Coding System
NIRS: Near-infrared spectroscopy
N-PASS: Neonatal Pain, Agitation, and Sedation Scale
TH: Therapeutic hypothermia
