# Supplementary material for: Interventions for the management of Pain and Sedation in Newborns undergoing Therapeutic hypothermia for hypoxic-ischemic encephalopathy (IPSNUT): protocol of a systematic review
Source: Syst Rev. 2022 May 23;11:101. doi: 10.1186/s13643-022-01982-9 (PMC9128112; doi:10.1186/s13643-022-01982-9)
Supplement: Supplementary file 2 — Additional file 2. Search Report. [file 13643_2022_1982_MOESM2_ESM.docx]

Search report

# project information

| request date | name | institution/organisation |
| --- | --- | --- |
| 20210120 | Matteo Bruschettini  Emma Olsson  Pyrola Bäcke  Ylva Thernström Blomqvist | Cochrane Sweden  Department of Pediatrics, Faculty of Medicine and Health, Örebro University, Örebro, Sweden  University Hospital, Neonatal Intensive Care Unit, Uppsala, Sweden  Department of Women's and Children's Health, Uppsala University, Uppsala, Sweden |

# project time frame – desired date of delivery

## January 2021

Search strategy peer reviewed for protocol April 2021

# describe research question and purpose with the search

The aim of the study is to perform a systematic review of pharmacological and non-pharmacological interventions for the management of pain and sedation in newborn infants undergoing therapeutic hypothermia for hypoxic-ischemic encephalopathy.

The review will be a Cochrane Review

# search terms

| Provide search terms according to pico(s), PEO or in free order |  |
| --- | --- |
| Patient / population / problem | Infants/neonates undergoing therapeutic hypothermia for hypoxic-ischemic encephalopathy |
| Intervention | Drug therapy, any dose, route, administration of  Morphine  Fentanyl  benzodiazepines  Midazolam  Alpha-2 agonists  Clonidine  Paracetamol  Non-pharmacological interventions: swaddling, sweet solutions, facilitated tucking and non-nutritive sucking |
| Comparison / control | See protocol |
| Outcome  Primary outcomes  1. Analgesia and sedation assessed with validated pain scales (the Neonatal Facial Coding system (NFCS) (Grunau 1987), the Neonatal Infant Pain scale (NIPS) (Lawrence 1993), the Premature Infant Pain Profile (PIPP and PIPP-r) (Stevens 1996, Stevens 2014 ), the Neonatal Pain, Agitation and Sedation scale (N-PASS) (Hummel 2008), the CRIES (acronym for Crying, Requires oxygen, Increased vital signs, Expressioni, Sleepless) scale (Krechel 1995), the Echelle Douleur Inconfort Nouveau-ne (EDIN) scale (Debillon 2001), the COMFORTneo (van Dijk 2009) and the Astrid Lindgren and Lund Children´s Hospital´s Pain and Stress Assessment scale for Preterm and Sick Newborn Infants (ALPS-neo) (Lundqvist 2014). Pain and sedation scores will be assessed during the 72 hours of the hypothermia procedure.  2. Circulatory instability, defined as hypotension requiring medical therapy (vasopressors, fluid boluses, or any changes in such drug administration).  3. Mortality to discharge.  4. Moderate-to-severe neurodevelopmental disability: cerebral palsy, developmental delay (Bayley Scales of Infant Development – Mental Development Index Edition II (BSID-MDIII; Bayley 1993; Bayley Scales of Infant and Toddler Development – Edition III Cognitive Scale (BSITD-III) (Bayley 2006); or Griffiths Mental Development Scale – General Cognitive Index (GCI) (GriPiths 1954; GriPiths 1970) assessment greater than two standard deviations (SDs) below the mean); intellectual impairment (intelligence quotient (IQ) greater than two SDs below the mean); blindness (vision less than 6/60 in both eyes) or sensorineural deafness requiring amplification (Jacobs 2013). We will separately assess data on children aged 18 to 24 months and aged three to five years.    Secondary outcomes  1. Neonatal mortality (death within 28 days of birth)  2. Duration of hospital stay  3. Days to reach full enteral feeding  4. Analgesia assessed with neurophysiological measures such as NIRS (near infrared spectroscopy) or GSR (galvanic skin response)  5. Focal gastrointestinal perforation  6. Episodes of bradycardia, defined as a fall in heart rate greater than 30% below baseline or less than 80 beats per minute for 10 seconds or longer, during exposure to the intervention  7. We plan to evaluate each of the components of “Moderate-to-severe neurodevelopmental disability” as a separate outcome and extract data on each long-term outcome from studies that evaluated children after 18 months’ chronological age, assessing data on children 18 to 24 months of age and on those 3 to 5 years of age separately. |  |
| Studytypes | Randomized studies |
|  | |

# references to key articles – examples of you want to have included in the search result (3-5 articles)

# desired delivery format (Endnote, word, pdf)

EndNote library

# Search strategies

Search strategy for protocol created 2021-01-20, peer reviewed and modified in April 2021.

No publication dates or language restrictions will be used.

## MEDLINE (Ebsco)

1. MH Asphyxia
2. MH Asphyxia Neonatorum
3. MH Hypoxia-Ischemia, Brain
4. MH Brain Ischemia
5. MH Hypoxia
6. MH Hypoxia, Brain
7. MH Brain Injuries
8. (brain injury or brain injuries)
9. (neuroprotect* or neuro-protect* or neuro-restorative or neurorestorative)
10. HIE
11. encephalopath*
12. (hypoxi* N2 ischaemi*)
13. (hypoxi* N2 ischemi*)
14. asphyxia*
15. (anoxi* N2 ischemi*)
16. (anoxi* N2 ischaemi*)
17. or/1-16
18. MH Hypothermia
19. MH Hypothermia, Induced
20. hypothermia*
21. cooling
22. 18 or 19 or 20 or 21
23. MH infant, newborn
24. TI (newborn* or new born or new borns or newly born or baby* or babies or premature or prematurity or preterm or pre term or low birth weight or low birthweight or VLBW or LBW or infant or infants or 'infant s' or infant's or infantile or infancy or neonat*) OR AB (newborn* or new born or new borns or newly born or baby* or babies or premature or prematurity or preterm or pre term or low birth weight or low birthweight or VLBW or LBW or infant or infants or 'infant s' or infant's or infantile or infancy or neonat*)
25. 23 or 24
26. PT randomized controlled trial
27. PT controlled clinical trial
28. AB randomized
29. AB placebo
30. drug therapy
31. AB randomly
32. AB trial
33. AB groups
34. or/26-33
35. MH animals not humans
36. 34 not 35
37. 25 and 36
38. TI randomi?ed or AB randomi?ed
39. TI randomly or AB randomly
40. TI trial or AB trial
41. TI groups or AB groups
42. TI ((single or doubl* or tripl* or treb*) and (blind* or mask*)) or AB ((single or doubl* or tripl* or treb*) and (blind* or mask*))
43. TI placebo* or AB placebo*
44. 38 or 39 or 40 or 41 or 42 or 43
45. 24 and 44

## CINAHLComplete (Ebsco)

1. (infant or infants or infantile or infancy or newborn* or "new born" or "new borns" or "newly born" or neonat* or baby* or babies or premature or prematures or prematurity or preterm or preterms or "pre term" or premies or "low birth weight" or "low birthweight" or LBW)

2.MH Hypoxia-Ischemia, Brain, Neonatal OR ( hypoxic-ischemic OR encephalopath* OR asphyxi* OR brain hypoxia OR brain injur* OR hypoxia neonatorum)

3.MH Hypothermia, Induced OR ( hypothermia OR induced hypothermia OR therapeutic hypothermia OR cooling)

4.PT randomized controlled trial OR PT controlled clinical trial OR ( randomized OR randomly OR randomised OR placebo OR drug therapy OR groups OR trial OR ) OR ( single OR doubl* OR tripl* OR treb*) AND (blind* OR mask*) )

5 1 AND 2 AND 3 AND 4 AND 5

## Cochrane CENTRAL

1 MESH DESCRIPTOR Asphyxia EXPLODE ALL AND CENTRAL:TARGET
2 MESH DESCRIPTOR Asphyxia Neonatorum EXPLODE ALL AND CENTRAL:TARGET
3 MESH DESCRIPTOR Hypoxia-Ischemia, Brain EXPLODE ALL AND CENTRAL:TARGET
4 MESH DESCRIPTOR Brain Ischemia EXPLODE ALL AND CENTRAL:TARGET
5 MESH DESCRIPTOR Hypoxia EXPLODE ALL AND CENTRAL:TARGET
6 MESH DESCRIPTOR Hypoxia, Brain EXPLODE ALL AND CENTRAL:TARGET
7 MESH DESCRIPTOR Brain Injuries EXPLODE ALL AND CENTRAL:TARGET
8 brain injury or brain injuries AND CENTRAL:TARGET
9 neuroprotect* or neuro-protect* or neuro-restorative or neurorestorative AND CENTRAL:TARGET
10 HIE AND CENTRAL:TARGET
11 encephalopath* AND CENTRAL:TARGET
12 hypoxi* ADJ2 ischaemi* AND CENTRAL:TARGET
13 hypoxi* ADJ2 ischemi* AND CENTRAL:TARGET
14 asphyxia* AND CENTRAL:TARGET
15 anoxi* ADJ2 ischemi* AND CENTRAL:TARGET
16 anoxi* ADJ2 ischaemi* AND CENTRAL:TARGET
17 #1 OR #2 OR #3 OR #4 OR #5 OR #6 OR #7 OR #8 OR #9 OR #10 OR #11 OR #12 OR #13 OR #14 OR #15 OR #16
18 MESH DESCRIPTOR Hypothermia EXPLODE ALL AND CENTRAL:TARGET
19 MESH DESCRIPTOR Hypothermia, Induced EXPLODE ALL AND CENTRAL:TARGET
20 hypothermia* AND CENTRAL:TARGET
21 cooling AND CENTRAL:TARGET
22 #18 OR #19 OR #20 OR #21
23 MESH DESCRIPTOR Infant, Newborn EXPLODE ALL AND CENTRAL:TARGET
24 infant or infants or infant's or "infant s" or infantile or infancy or newborn* or "new born" or "new borns" or "newly born" or neonat* or baby* or babies or premature or prematures or prematurity or preterm or preterms or "pre term" or premies or "low birth weight" or "low birthweight" or VLBW or LBW or ELBW or NICU AND CENTRAL:TARGET
25 #24 OR #23
26 #17 AND #22 AND #25
27 2007 TO 2020:YR AND CENTRAL:TARGET
28 #27 AND #26

## ISRCTN

Date ranges: 01 January 2007 to 26 November 2020

Terms:

Hypoxia AND ( Interventions: Cooling AND Participant age range: Neonate )

Ischemia AND ( Interventions: Cooling AND Participant age range: Neonate )

Asphyxia within Interventions: Cooling AND Participant age range: Neonate

Condition: Encephalopathy AND Interventions: Cooling AND Participant age range: Neonate

Condition: Encephalopathy AND Interventions: Hypothermia AND Participant age range: Neonate

Condition: Hypoxia AND Interventions: Hypothermia AND Participant age range: Neonate

Condition: Ischemia AND Interventions: Hypothermia AND Participant age range: Neonate

Condition: Asphyxia AND Interventions: Hypothermia AND Participant age range: Neonate
